# The Link Between Employees’ Sense of Vitality and Proactivity: Investigating the Moderating Role of Personal Fear of Invalidity

**DOI:** 10.3389/fpsyg.2020.02169

**Published:** 2020-09-08

**Authors:** Burkhard Wörtler, Nico W. Van Yperen, Jesús M. Mascareño, Dick P. H. Barelds

**Affiliations:** Department of Psychology, University of Groningen, Groningen, Netherlands

**Keywords:** proactive work behavior, work performance, anxiety, affect, well-being, mental energy, experimental study

## Abstract

Proactive behavior has emerged as a key component in contemporary views of individual work performance. Hence, a central question in the literature is how to enhance employees’ proactive behavior. We investigated whether the more that employees experience a sense of vitality (i.e., energizing positive affect), the more likely they are to show proactive behavior at work, and whether this applies only to employees with a low personal fear of invalidity [(PFI) i.e., the inclination to be apprehensive about the risks/negative consequences of making errors]. Experimental (*N* = 354) and cross-sectional field (*N* = 85) studies provided consistent evidence for a positive relation between employees’ sense of vitality at work and their self-rated proactivity. The predicted moderation effect was observed only for manager-rated proactivity. We conclude that feeling energized in the workplace is not necessarily associated with observable proactive behavior. It is only when employees experiencing a sense of vitality at work are not prone to fearing the risks/negative consequences of making errors that they are more likely to show observable proactive behavior in an organization.

## Introduction

In today’s dynamic workplaces, proactive behavior has emerged as a key component of individual job performance (Crant, 2000; Griffin et al., 2007; Bindl and Parker, 2011) and as a topic of great relevance for organizational research (Parker and Bindl, 2016). Proactivity at work has been described as “taking initiative in improving current circumstances or creating new ones; it involves challenging the status quo rather than passively adapting to present conditions” (Crant, 2000, p. 436). In addition to the proactive personality concept (Bateman and Crant, 1993), various concepts of proactive behavior have been used in the organizational sciences (Parker and Collins, 2010). A prevalent concept is that of personal initiative, which describes self-starting actions aimed at changing the work situation to enhance the status quo (Frese et al., 1997; Fay and Frese, 2001). A closely related concept (see Fay and Frese, 2001) is that of taking charge, which refers to change-related efforts to enhance organizational functioning by improving work methods and procedures (Morrison and Phelps, 1999).

Since proactivity has the potential to benefit organizations, teams, and individual employees, a central question in the literature is how employees’ proactivity can be enhanced (Parker et al., 2006; Bindl and Parker, 2011; Strauss and Parker, 2018). Drawing on the model of proactive motivation developed by Parker et al. (2010), we posit that such forms of proactive behavior vary as a function of employees’ sense of vitality at work. Vitality has been referred to as “the positive feeling of having energy available to oneself” (Nix et al., 1999, p. 266; see also Ryan and Frederick, 1997).

The first aim of the present research was to provide *initial* evidence that the link between work-related vitality and employee proactivity (e.g., Binyamin and Brender-Ilan, 2018) is of a causal nature. A core tenet of the model of proactive motivation is that activated positive affect (i.e., vitality) is an *antecedent* of proactivity at work (Parker et al., 2010). However, empirical support to date for the link between vitality and proactive behavior stems from studies relying on cross-sectional (Binyamin and Brender-Ilan, 2018) and diary research (Schmitt et al., 2017) designs. Such non-experimental research does not preclude the possibility that this relation is spurious; that is, it exists due to third variables (e.g., Spector, 2019). Furthermore, proactive behavior was found to predict employees’ sense of vitality (see Cangiano et al., 2019). To claim that vitality indeed increases employee proactivity, Bindl et al. (2012) and Schmitt et al. (2017) called for experimental evidence for this relation. As MacKinnon et al. (2012, p. 4) put it, “Random assignment of subjects to experimental conditions is the gold standard for making causal inference about the relationship between two variables.” We have therefore sought to complement previous research by employing a study using a randomized controlled design (Study 1).

Furthermore, drawing on Conservation of Resources Theory (Hobfoll, 1989), our second aim was to demonstrate that employees’ personal fear of invalidity (PFI) moderates the relation between vitality and proactive behavior (Study 1 and Study 2). PFI is a personality/individual-difference variable that refers to an individual’s inclination to be apprehensive about making errors, wrong choices, or judgments and to worry about the risks/negative consequences of his or her decisions (Thompson et al., 2001). We will argue that a strong PFI will counteract the effect of vitality on proactive behavior because such behavior involves deliberate decision-making and carries social risk (e.g., Morrison and Phelps, 1999). Evidence for such a moderating role of PFI is a valuable contribution to the literature on work-related proactivity because there is a lack of research on the boundary conditions of the relation between vitality and proactivity (for an exception, see Schmitt et al., 2017). It is rather unlikely that all employees are more inclined to be proactive when experiencing a sense of vitality. Instead, individual differences are likely to alter the relation between positive affect at work and employees’ behavioral responses (e.g., Ilies et al., 2006). Before we discuss the presumed moderating role of PFI, we first explain the link between vitality and proactivity.

### Proactive Behavior Resulting From a Sense of Vitality at Work

Behavior is proactive when it is “future-focused,” “changed oriented,” and “self-starting” (Parker and Bindl, 2016, pp. 1–2). Proactive behavior is discretionary (Morrison and Phelps, 1999; Belschak and Den Hartog, 2010) and can have various desirable consequences for organizations, teams, and individual employees (Bindl and Parker, 2011). For example, individuals with a proactive personality tend to have an advantage in terms of career success and employability (Fuller and Marler, 2009). Fuller et al. (2012) showed that in-role performance ratings of employees tended to be higher when they showed proactive behavior, particularly when their supervisors had a proactive personality. In the present study, we expected that employees’ sense of on-the-job vitality would increase their proactive behavior at work. Individuals experiencing a sense of vitality possess mental energy and vigor, whereas those lacking vitality feel exhausted (Ryan and Frederick, 1997; Nix et al., 1999). Having enthusiasm for one’s activities is a key feature of those experiencing a sense of vitality (Ryan and Bernstein, 2004).

Based on joint consideration of the model of proactive motivation in the work domain (Parker et al., 2010) and the conceptualization of vitality, it seemed reasonable to assume that employees’ sense of vitality would increase their likelihood of showing proactive behavior at work. Parker et al. (2010) have identified proactivity as a goal-driven process and have viewed *activated* positive affect, among other things, as a core motivational state propelling proactive goal striving (see also Bindl et al., 2012). It has been argued that proactive behavior takes effort as energy is required in all phases of bringing about change (Grant and Ashford, 2008; Bolino et al., 2010). Sensing vitality is a conscious experience (Ryan and Frederick, 1997) of having energy available that one can harness or regulate for purposive actions (Ryan and Deci, 2008). The activation inherent in having energy available is what distinguishes a sense of vitality from other positive feelings (Nix et al., 1999; Ryan et al., 2010). A key feature of individuals who experience a sense of vitality is the use of energy and enthusiasm to fuel their own activity and productivity (Ryan and Bernstein, 2004). In a related vein, Parker et al. (2010) claim that *activated* positive affect represents an energizing motivational state that is essential to proactive goal striving because activation increases the effort put into actions. Christian et al. (2011) showed that employees who experience a sense of work-related mental vigor (i.e., vitality) are, among other things, more inclined to show discretionary job performance. According to these authors, this may be due to the considerable amount of mental resources available to such employees, which they can use to pursue unrequired job activities that tend to demand mental resources.

Previous empirical findings also justify predicting a positive relation between employees’ energizing sense of vitality and their proactivity (Schmitt et al., 2017; Binyamin and Brender-Ilan, 2018). Vitality is positively associated with physical health (Ryan and Frederick, 1997), which is important for behaving proactively in the first place. Furthermore, studies have revealed a positive relation between an energizing affective-motivational state of mind (Schaufeli et al., 2002) and proactivity (e.g., Salanova and Schaufeli, 2008; Schmitt et al., 2016). Similarly, other research has found that positive affect is positively related to proactive behavior (Fay and Sonnentag, 2012), including day-level taking charge behavior (Fritz and Sonnentag, 2009), task proactivity (Bindl et al., 2012), and issue implementation (Sonnentag and Starzyk, 2015). Based on these indications, in the present research, we therefore hypothesized that:

*Hypothesis 1*. A positive relation exists between employees’ sense of vitality at work and their proactive behavior.

### The Moderating Effect of Personal Fear of Invalidity

PFI is an affective trait referring to an individual’s inclination to be apprehensive about the risks/negative consequences of making errors. Individuals with a strong PFI tend to struggle with making decisions due to the possibility of being wrong (Thompson et al., 2001). A pronounced PFI may counteract the propensity of employees high in vitality to show proactive behavior such that the likelihood of those employees showing proactive behavior is no higher than among employees who lack a sense of vitality at work.

Central to our rationale for the proposed moderation effect of PFI is the idea that proactivity involves a calculated, deliberate decision-making process (Morrison and Phelps, 1999; Parker et al., 2006). This may involve an evaluation of the consequences and outcomes of being proactive given that the impact is not necessarily foreseeable (Parker et al., 2010). Whereas proactivity is meant to improve the status quo (Parker et al., 2010), engaging in proactive behavior can involve uncertainty and social risk if proactivity is not welcome by others (Fay and Frese, 2001; McAllister et al., 2007; Wu and Parker, 2017). Specifically, coworkers and superiors may have doubts about changes to the status quo (Morrison and Phelps, 1999; Fay and Frese, 2001) since proactivity may involve “disrupting or deviating from assigned tasks, prescribed roles, reified norms, accepted practices, and existing routines” (Grant and Ashford, 2008, p. 24). Thus, employees may choose not to show proactive behavior owing to their fear of the consequences (e.g., Kish-Gephart et al., 2009). Based on those features of proactive behavior, we suggest that, for two reasons, a marked PFI is likely to counteract the proclivity of employees experiencing a sense of vitality to show proactive behavior.

First, PFI is likely to offset the energy available for proactive actions in employees experiencing a sense of vitality. The dual process approach to information processing (see, for example, Fennis and Stroebe, 2010) posits that judgment and decision-making are positioned on a continuum from automatic top-down processing to more controlled (bottom-up) processing. As a controlled processing style is effortful and requires mental resources (Fennis and Stroebe, 2010), it is likely to deplete energy. The controlled processing style is more likely to be adopted by individuals high in PFI when deciding whether to show proactive behavior because they are less likely to use heuristic processing and avoid reaching conclusions quickly (Thompson et al., 2001). Research has shown that anxiety is negatively associated with performance through feeling exhausted emotionally (McCarthy et al., 2016) and also that anxiety increases rumination, which decreases the likelihood of employees engaging in helping behaviors, possibly because rumination depletes employees’ energy (Calderwood et al., 2018). These findings indirectly support our rationale that a strong PFI will counteract employees’ energy for proactive behavior because anxiety regarding decision-making is heightened in individuals high in PFI.

Second, employees who experience a sense of vitality at work and have a strong PFI may deliberately choose not to engage in proactive behavior in order to avoid stress and energy depletion. Indeed, the central proposition of Conservation of Resources Theory (Hobfoll, 1989) is that “people strive to retain, protect, and build resources and that what is threatening to them is the potential or actual loss of these valued resources” (p. 516). Individuals with a marked PFI tend to experience high levels of “predecisional conflict,” involving scanning for potential overlooked negative consequences to avoid regret after having committed to a decision (Thompson and Zanna, 1995, p. 266). Such predecisional conflict, which is likely to be experienced by employees high in PFI when contemplating a proactive behavior, is conceivably stressful and energy-draining. Based on the conservation of resources perspective, we surmise that a strong PFI would lead employees who possess positive mental energy (i.e., experience a sense of vitality) to refrain from proactive behavior in order to avoid the energy loss involved in predecisional conflict. This would enable those employees to conserve their energy at work, potentially resulting in them prioritizing more default types of activities, such as working on tasks that are contractually required of them (Christian et al., 2011).

Empirical support for our assumption that employees high in PFI are likely to refrain from showing proactive behavior even when they feel energized to do so stems from a qualitative study conducted by Bindl (2019). While positive and negative discrete emotions can motivate proactivity (Parker et al., 2010; Sonnentag and Starzyk, 2015), Bindl (2019) found that fear is a key discrete emotion that can thwart the implementation of a proactive behavior by someone initially motivated so to perform. For example, employees may not proactively implement a change because of their anxiety about the reaction, such as disapproval, this could evoke from others (Bindl, 2019). This emphasizes the importance of focusing on the role of PFI rather than general negative affective states, such as workplace anxiety (McCarthy et al., 2016), as a moderator of the link between work-related vitality and proactivity: High PFI individuals are concerned about the perceived risk of their judgments and actions, so their anxiety is focused on the outcomes of their decisions (Thompson et al., 2001). On this basis, we propose that employees high in PFI may not respond to a sense of vitality by showing proactive behaviors. This leads to the following hypothesis:

*Hypothesis 2.* Personal fear of invalidity moderates the positive relation between employees’ sense of vitality at work and proactive behavior such that this positive relation exists only if personal fear of invalidity is low.

## Study 1

Given that we aimed to show that the link between work-related vitality and proactive behavior is of a causal nature, we first tested our hypotheses in an experimental study using a randomized controlled design. This involved measuring participants’ PFI and situationally inducing a sense of vitality in them through a manipulation before assessing their proclivity to behave proactively in a variety of hypothetical work situations.

### Materials and Methods

#### Participants and Design

We recruited participants using Amazon Mechanical Turk (MTurk), an online crowdsourcing platform. MTurk samples are seen as comparable in quality to other convenience samples including organizational samples (Buhrmester et al., 2011; Landers and Behrend, 2015). The participants were given $1.50 compensation for their time. We first calculated the sample size required to detect a small- to medium-sized effect, at a statistical power of 0.80 and a statistical significance level of 0.05, based on Cohen’s (1992) recommendations. We concluded that 300–350 participants were required for our study. Anticipating the removal of some cases during data screening (see below), we recruited a total of 413 individuals. The study was approved by the Ethics Committee of Psychology of the University of Groningen, and participants gave their informed consent.

We followed recommendations for obtaining good quality data by using system qualifications pertaining to location and reputation (Peer et al., 2014; Keith et al., 2017). That is, we only recruited employees from the US who had had at least 50 tasks approved on MTurk and a high ratio (above 97%) of approved vs. submitted tasks. To recruit a sample comparable to an organizational convenience sample and to avoid a sample comprising professional MTurk users who consider completing tasks on MTurk as their primary job (Keith et al., 2017), we included a further system qualification: Participants had to be employed full-time (i.e., for 35 or more hours per week). To further ensure good data quality, we removed cases from the data set if the participants indicated at the end of the study that they (a) were not honest in all responses (*n* = 7), (b) randomly responded to items (*n* = 11), (c) did not complete the study without an interruption (*n* = 10), and/or (d) did not put effort into the specific task that represented the experimental manipulation (*n* = 5), which is described below. Additional criteria for removing respondents were not providing the correct response to an attentiveness check (*n* = 18) and incorrectly responding to an instructed response item (*n* = 18; see Meade and Craig, 2012). Some of the removed cases failed more than one of the criteria.

The final sample comprised 354 employees (63% of whom were women), who ranged in age between 21 and 64 years and worked in their current position (i.e., job tenure) between less than 1 year and 33 years. The majority of the participants (60%) were in a leadership position. In terms of the highest education level achieved, almost half of the participants (47%) held a bachelor’s degree, followed by a master’s degree (16%), college education but no degree (16%), associate degree in college (12%), high school diploma or equivalent (4%), professional degree such as JD or MD (3%), and doctoral degree (1%). The participants worked in a variety of industries, of which the healthcare and social assistance (17%) and the financial and business consultancy (12%) sectors were the most prevalent.

We used a one-factor (vitality: high or low vs. control) between-subjects design. The participants were randomly assigned to one of the three experimental conditions. The high-vitality condition had 124 participants, and the low-vitality condition and control conditions each had 115 participants.

#### Procedure, Manipulation, and Materials

The participants accessed the study through a hyperlink provided on MTurk. The survey software *Qualtrics* randomly assigned the participants to one of the three experimental conditions. All participants provided sociodemographic information and completed a self-reporting measure assessing their PFI prior to being administered the experimental manipulation of work-related vitality. After the manipulation, the participants completed a measure of proactive behavior, a manipulation check, and an attentiveness check before, finally, evaluating their response behavior. We describe the manipulation and measures below.

##### Vitality (Manipulation)

We manipulated work-related vitality using a self-developed experiential prime (see Appendix) based on an established experiential prime paradigm (see Galinsky et al., 2003). All participants were asked to recall and relive a workday and to describe as vividly and in as much detail as possible what happened on that day at work and what they thought and did. The participants in the high-vitality condition were asked to recall and relive a workday on which they had experienced a sense of vitality at work. Based on the conceptualization of vitality adopted (Ryan and Frederick, 1997; Ryan and Bernstein, 2004; Ryan and Deci, 2008), we explained that by experiencing a sense of vitality, we meant having a lot of energy and enthusiasm at work. One participant, for example, recalled a recent busy day on which some colleagues had been stressed out. However, that participant remembered being in total control and having so much energy that day that he had been able to help all the other employees with their work.

The participants in the low-vitality condition were asked to recall a workday in which they had not experienced a sense of vitality at work. We specified this by stating that we were referring to a lack of energy and enthusiasm at work. For example, one participant recalled a day that just seemed off for him. He was tired even though he had got enough sleep the night before. Everything he did seemed to take extra effort. By the end, that participant just wanted to go home and “crash out” on the bed.

The control condition represented a neutral group in which neither a high nor a low sense of vitality was salient. Specifically, the participants in the control condition were asked to recall a typical workday. For instance, one participant told that when she enters the office she first logs on to her computer. She then begins her day by making a list of all the records that had been requested the previous day. After that, she fills in forms and ensures records are copied and delivered to those who have requested them.

##### Manipulation Check

To assess participants’ sense of vitality on the workday they recalled, we used the five-item vitality-at-work subscale developed by Porath et al. (2012), which we adapted to the past tense. We instructed the participants to refer to the workday they had recalled and written about when providing their responses. An example item is: “On that workday, I had energy and spirit at work.” The participants used a response scale ranging from (1) *strongly disagree* to (7) *strongly agree*. We averaged the item scores into an overall score (α = 0.97).

##### Proactive Behavior

We used the Situational Judgment Test of Personal Initiative (SJT-PI; Bledow and Frese, 2009) as a measure of proactive behavior (see also Wu et al., 2018). The SJT-PI comprises 12 items, each of which describes a hypothetical but realistic situation that could occur in the workplace. Four or five response options are provided for each item. Each response option can be rated as “most likely” or “least likely.” For each item, one response option must be chosen by the responder as the “most likely” response and another as the “least likely” response. Thus, *two* response options are selected for each item. As an example, one situation describes team meetings organized by a supervisor that are perceived as unsatisfactory due to their inefficient structure and to digressions from the main topic during the meetings. The supervisor would not, however, see any reason for change and would be irritated if criticized. A response option that reflects high proactivity is to take charge and organize the team meetings more effectively. Accepting the situation as it is and making the best of it is a response option reflecting low proactivity. When presenting the items to the participants, we asked them to consider the workday they had previously recalled and written about. A great advantage of using the SJT-PI is that it is less susceptible to socially desirable responding than Likert-scale types of measures, which can be a cause for concern with self-reported data (Paulhus, 2002). The SJT-PI is less susceptible to this type of response bias because response options that do not reflect proactivity describe reasonable behavior (Bledow and Frese, 2009).

Responses that reflect high proactivity chosen as the most likely response and responses reflecting low proactivity chosen as the least likely response were each scored as one. Response options reflecting low proactivity chosen as the most likely response and response options reflecting high proactivity chosen as the least likely response were each scored as minus one. All other responses were scored as zero. The total score on each item could, therefore, vary on a five-point scale from -2 to 2, with higher scores implying that the situation described would be dealt with more proactively. A participant’s overall score was the average of the 12 item scores (α = 0.75).

##### Personal Fear of Invalidity

We used four items from the PFI scale developed by Thompson et al. (2001). We selected the four items that Thompson et al. (2001) found to have the highest factor loadings (ranging from 0.68 to 0.73). An example item is: “I can be reluctant to commit myself to something because of the possibility that I might be wrong.” The participants used a response scale ranging from (1) *strongly disagree* to (7) *strongly agree*, and the item scores were averaged into an overall score (α = *0.87*).

#### Statistical Analysis

We performed a hierarchical multiple regression analysis to test both hypotheses. We converted the vitality factor into two dummy variables, referred to here as d_1_ and d_2_. We coded the low-vitality condition as the reference category, allowing a comparison of the means of the high-vitality and the low-vitality conditions. This mean difference is labeled d_1_. The mean difference between the control condition and the low-vitality condition is labeled d_2_. We multiplied each of the two dummy variables by mean-centered PFI to create the interaction terms (Cohen et al., 2003). Since the variable leadership position was significantly related to proactivity (see below), we included this variable as a covariate in the first step of the regression analysis to increase the precision of the regression estimates. We entered the two dummy variables and mean-centered PFI in the second step and the two interaction terms in the third step.

### Results

#### Manipulation Check

We performed a one-way analysis of variance (ANOVA) to assess the outcome of the manipulation, and this revealed a significant effect, *F*(2, 351) = 342.78, *p* < 0.001, η^2^*_*p*_* = 0.66. Further, as the Levene’s test indicated a violation of the homogeneity of variance assumption, *F*(2, 351) = 15.57, *p* < 0.001, we additionally performed a Welch’s test, which confirmed a significant difference between at least two of the conditions in the mean score of the manipulation check variable, Welch’s *F*(2, 213.89) = 427.46, *p* < 0.001. Planned comparisons that did not assume equal variances in the conditions revealed that participants in the high-vitality condition scored significantly higher (*M* = 6.09, *SD* = 0.80) on the manipulation check variable than participants in the low-vitality condition (*M* = 2.18, *SD* = 1.21), *t*(196.36) = 29.27, *p* < 0.001 and in the control condition (*M* = 4.83, *SD* = 1.44), *t*(175.50) = 8.24, *p* < 0.001. Similarly, the participants in the low-vitality condition scored significantly lower on the manipulation check variable than the participants in the control condition, *t*(221.00) = -15.12, *p* < 0.001. From this, we concluded that our manipulation of vitality worked as intended.

#### Bivariate Associations and Group Comparisons

Participants in a leadership position scored significantly higher on proactive behavior (*M* = 0.17, *SD* = 0.67) than those who were not (*M* = −0.31, *SD* = 0.59), *t*(352) = 6.93, *p* < 0.001. Consequently, we decided to include the leadership position variable in the analysis when testing the hypotheses. The correlations among the continuous variables are presented in Table 1. This shows that PFI was significantly negatively correlated with proactive behavior.

**TABLE 1:** Means, standard deviations, and correlations of the variables in Study 1.

Variable	*M*	*SD*	2	3	4
1. Proactive behavior	–0.02	0.68	–0.15	0.03	0.02
2. Personal fear of invalidity	3.69	1.44	–	–0.18	–0.10
3. Age	38.92	9.80		–	0.45
4. Job tenure	6.01	5.47			–

*N = 354. Correlations above 0.10 and 0.14 (in absolute values) are significant at the p = 0.05 and p = 0.01 level, respectively.*

#### Hypothesis Testing

**FIGURE 1:**
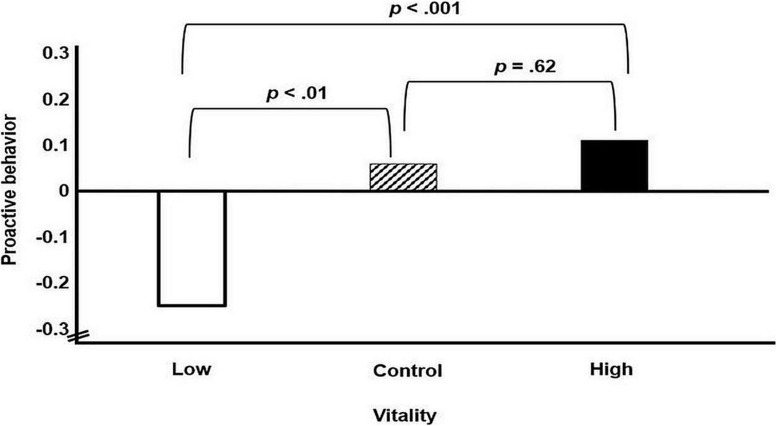
Main effect of work-related vitality on employees’ proactive behavior in Study 1. “Low,” “control,” and “high” refer to the experimental conditions of manipulated work-related vitality.

In the first hypothesis, we proposed a positive relation between employees’ sense of vitality at work and their proactive behavior. The results are shown in Figure 1. The mean proactive behavior score was higher in the high-vitality condition (*M* = 0.11, *SD* = 0.60) than in the low-vitality condition (*M* = −0.25, *SD* = 0.74). As shown in Table 2, the slope corresponding to that mean difference (as represented by the dummy variable d_1_) was significantly different from zero, 95% CI [0.16, 0.48]. This result confirmed our first hypothesis.

**TABLE 2:** Summary of hierarchical regression analysis for variables predicting proactive behavior in Study 1.

	Model 1			Model 2			Model 3		
Variable	*B*	*SE*_*B*_	β	*B*	*SE*_*B*_	β	*B*	*SE*_*B*_	β
Leadership position^*a*^	0.48***	0.07	0.35	0.45***	0.07	0.33	0.45***	0.07	0.32
High vitality–Low vitality (d_1_)^*b*^				0.32***	0.08	0.23	0.32***	0.08	0.23
Control–Low vitality (d_2_)^*c*^				0.28**	0.08	0.19	0.28**	0.08	0.20
Personal fear of invalidity				–0.04	0.02	–0.09	–0.04	0.04	–0.08
d_1_ × Personal fear of invalidity							–0.01	0.06	–0.02
d_2_ × Personal fear of invalidity							0.01	0.06	0.01
Intercept	−0.31***	0.05		−0.50***	0.07		−0.50***	0.07	
*F*_change_	48.00			7.58			0.07		
*R*^2^	0.12***			0.17***			0.17***		
Δ*R*^2^	0.12***			0.05***			0.00		

N = 354. **p < 0.01. ***p < 0.001. ^a^Leadership position was coded 1 = yes and 0 = no (reference level). ^b^Dummy variable d_1_ expresses the mean difference between the high-vitality condition and low-vitality condition. ^c^Dummy variable d_2_ expresses the mean difference between the control condition and low-vitality condition.

Furthermore, the mean proactive behavior score in the control condition (*M* = 0.06, *SD* = 0.65) was significantly higher than that in the low-vitality condition (*M* = −0.25, *SD* = 0.74) with again a significant slope (95% CI [0.12, 0.44]) corresponding to the dummy variable d_2_. We also checked whether the high-vitality condition and the control condition differed significantly in proactive behavior by using the control condition as the reference category; here, the mean difference did not predict proactive behavior, *b* = 0.04, *t*(349) = 0.50, *p* = 0.62, 95% CI [−0.12, 0.20].

In the second hypothesis, we proposed that PFI moderated the positive relation between employees’ sense of vitality at work and their proactive behavior such that this positive relation existed only if PFI was low. As shown in Table 2, the results did not support an interactive effect between vitality and PFI on proactive behavior. The mean difference between the high-vitality condition and the low-vitality condition did not vary as a function of PFI, 95% CI [−0.12, 0.09], and, thus, our second hypothesis was rejected.

## Study 2

Study 1 indicated a positive relation between work-related vitality and proactivity irrespective of PFI. However, the measurement of proactive behavior in Study 1 required participants to respond to hypothetical scenarios. Moreover, the findings are based on only self-reported proactivity. Given these concerns, and noting Crandall and Sherman’s (2016) observation that “conceptual replications are critical for establishing the generalizability of an initial observation and the theory it purports to support” (p. 94), we conducted a field study (Study 2) as a conceptual replication to address these limitations.

In our conceptual replication, we used a different concept of proactive behavior: taking charge (Morrison and Phelps, 1999). Both concepts, personal initiative (Study 1) and taking charge (Study 2), have been used in previous studies of proactive behavior (Tornau and Frese, 2013). Fay and Frese (2001, p. 112) concluded that “There is a large overlap of PI [personal initiative] and taking charge.” As we discuss in the *Introduction*, taking charge similarly refers to proactive behaviors that are voluntary and functional/constructive; that is, they are meant to benefit the organization (Fay and Frese, 2001). We used the concept of taking charge in Study 2 for three reasons. First, utilizing the SJT-PI used in Study 1 is too time-consuming in the context of a field study and would deter organizations from participating. In contrast, employees’ charge-taking behavior can be measured time efficiently by both self-reporting and through others’ reports (Morrison and Phelps, 1999; Parker and Collins, 2010). Second, the SJT-PI cannot be used to obtain manager ratings of employees’ proactivity. Third, adopting a related, but different, concept of proactive behavior could expand the scope of our findings.

### Materials and Methods

#### Participants

We recruited 98 employees from an outsourcing and payroll management company in Mexico. The employees worked in one of 11 departments, each of which was led by one manager. Of the participants who provided sufficient data to be included in the analysis (*n* = 96), we omitted the responses of a further five who did not provide the correct response to an instructed response item that was included to detect careless responding (Meade and Craig, 2012) and/or indicated that they provided random responses to some of the survey items (*n* = 4). One participant is represented twice here: in addition to having provided an incorrect response to the instructed response item, this participant admitted to having responded randomly. Moreover, we identified three participants who took the survey twice. In each case, we removed the second submission.

The final sample therefore comprised 85 participants (62 women, 23 men), ranging in age from 22 to 58 years. Most participants worked 48 hours per week according to their labor contract (86%) and had a university degree (87%). The participants’ job tenures ranged from less than a year to 10 years. Most employees reported that they had contact with their department manager very often (31%), often (37%), or occasionally (32%) on a typical day. Only one employee rated the contact to be rare, and none indicated having no contact at all on an ordinary day.

In addition to these participants, we involved all 11 department managers (seven women, four men; ranging in age from 33 to 52; *M*_age_ = 40.09, *SD*_age_ = 6.04). Managers rated the proactive behavior by all the employees in their department (ranging from 2 to 20). As such, our data have a multilevel structure. Employees (level-one units) were nested in departments (level-two units). The participants did not receive compensation for their participation, but they were all promised a report on the findings, including an oral presentation of the findings to the department managers. The study was approved by the Ethics Committee of Psychology of the University of Groningen, and participants gave their informed consent.

#### Materials

The measures were part of a more general survey on occupational well-being and proactive behavior at work. One of the authors, a bilingual native Spanish speaker from Mexico, translated the English items into Spanish after conferring with the other authors about the meaning of the original English items.

##### Vitality

There is consensus in the literature that vitality is a “phenomenologically accessible and salient” experience that can be appropriately assessed through self-report questionnaire (Ryan and Bernstein, 2004, p. 275; see also Chan, 2009). We used the five-item vitality-at-work subscale developed by Porath et al. (2012). This scale was also used for the manipulation check in Study 1. A sample item is “At work, I have energy and spirit.” The participants used a response scale ranging from (1) *strongly disagree* to (7) *strongly agree*. Based on the item analysis, we decided to exclude the only reversed item because its scores correlated rather poorly with the total score from that scale (*r*_item__–to__tal_ = 0.24; see also Bostic et al., 2000). Removing this item increased the estimated scale reliability from α = 0.80 to α = 0.95.

##### Proactive behavior

We asked each department manager to rate his or her subordinate employees’ proactive behavior by responding to a four-item “taking charge” measure (α = 0.92 in the current study; Parker and Collins, 2010; see also Morrison and Phelps, 1999). A sample item is “This employee tries to implement solutions to pressing organizational problems.” However, not all behaviors are visible to others (Bergeron, 2007), and employees’ and managers’ proactive behavior ratings will be based on different information and perspectives (Tornau and Frese, 2013). Therefore, we also measured employees’ self-reported proactive behavior. To measure employees’ self-reported proactive behavior, we used the same items, though phrased in the first-person perspective (with a resulting α = 0.85). Both managers and subordinate employees used a seven-point response scale ranging from (1) *never* to (7) *always*.

##### Personal fear of invalidity

We used the same items as in Study 1 for assessing employees’ PFI (α = 0.71).

#### Statistical Analysis

Employees (level-one units) within the same department (level-two unit) will share influences (e.g., the work environment) that may make them more similar to each other than to employees in other departments. Data are therefore likely to be more similar among participants of the same department than with participants from other departments. This violates the assumption of independence of observations that is central to ordinary least squares linear regression analysis (Snijders and Bosker, 2012). Consequently, we performed separate multilevel regression analyses for self-reported and for manager-reported proactive behavior (Snijders and Bosker, 2012) using the *nlme* package (Pinheiro et al., 2020) developed for software package R (R Core Team, 2019) to test the hypotheses.

Expecting an interactive effect between vitality and PFI, we grand mean centered these variables to facilitate interpretation and remove the risk of multicollinearity induced by the inclusion of main effects and an interaction effect in the same model (Cohen et al., 2003). We followed recommendations for testing fixed effects in small samples by relying on standard errors produced by restricted maximum likelihood (REML) estimations (Snijders and Bosker, 2012). Using REML, we first specified an empty model without predictors (i.e., a null model) to examine how the variance in taking charge was portioned into level-one and level-two variances. Subsequently, we fitted a model that included participants’ sex as a control variable (see below), as well as vitality and PFI to test *Hypothesis 1*. Finally, we added the interaction effect of vitality and PFI to test *Hypothesis 2*. We did not estimate a random slope multilevel model, which would imply estimating a separate regression line for each department, due to the low number of level-two units (i.e., departments) in our sample. We used the deviance test to compare the fit of nested models (Snijders and Bosker, 2012; Finch et al., 2014). The deviance test assesses whether adding predictor variables to a model results in a statistically significant improvement in model fit. The deviance test has a chi-squared distribution with degrees of freedom equal to the number of added parameters. We considered a *p* < 0.05 to be sufficient evidence that the larger model provided an improved fit over the simpler model. In order to carry out deviance tests, we were required to specify all the models again using maximum likelihood estimation to obtain accurate comparisons (see Snijders and Bosker, 2012; Finch et al., 2014).

### Results

#### Descriptive Statistics and Correlations

Means, standard deviations, and correlations among the continuous variables are presented in Table 3. Vitality correlated positively and significantly with self-rated proactive behavior, but the correlation with manager-rated proactive behavior was not significant. It is also noteworthy that self-ratings and manager ratings of proactive behavior were not significantly correlated.

**TABLE 3:** Means, standard deviations, and correlations of the variables in Study 2.

Variable	*M*	*SD*	2	3	4	5	6	7	8
1. Proactive behavior (Self-rated)	5.16	1.09	–0.09	0.34	–0.14	0.04	0.22	0.00	0.04
2. Proactive behavior (Manager-rated)	5.37	1.06	–	0.12	–0.04	–0.14	0.11	0.16	–0.05
3. Vitality	5.99	0.98		–	–0.21	0.27	–0.01	0.19	0.12
4. Personal fear of invalidity	2.87	1.10			–	–0.19	–0.08	–0.07	–0.09
5. Age	31.56	7.85				–	0.11	0.19	0.31
6. Work hours	45.65	8.84					–	0.03	0.02
7. Daily contact with manager	3.96	0.82						–	0.06
8. Job tenure	2.13	2.24							–

*N = 85. Correlations above 0.21 and 0.28 (in absolute values) are significant at the p = 0.05 and p = 0.01 level, respectively.*

There was a tendency for the managers to rate women as showing proactive behavior more frequently (*M* = 5.50, *SD* = 1.04) than their male counterparts (*M* = 5.04, *SD* = 1.07), *t*(83) = 1.77, *p* = 0.08. Therefore, and in line with previous research on taking charge (e.g., Fuller et al., 2012; Li et al., 2016), we included participants’ sex as a control variable. The proportion of the total variance in self-rated proactive behavior and manager-rated proactive behavior explained by department belonging [in terms of the intraclass correlation coefficient (ICC)] was 0.15 and 0.14, respectively.

#### Hypothesis Testing

The results of the multilevel regression analysis concerning self-rated and manager-rated proactive behavior are shown in Tables 4, 5, respectively. In the first hypothesis, we posited a positive relation between employees’ sense of vitality at work and their proactive behavior. As indicated by the zero-order correlation presented in Table 3, the regression results confirmed a positive relation between vitality and self-rated proactive behavior, 95% CI [0.12, 0.58]. However, there was no evidence for a positive relation between vitality and manager-rated proactive behavior, 95% CI [−0.05, 0.41]. Thus, our first hypothesis was only confirmed for self-rated proactive behavior.

**TABLE 4:** Summary of the multilevel regression analysis for variables predicting self-rated proactive behavior in Study 2.

	Null model		Main effect model		Model with interaction	
	γ	*SE*	γ	*SE*	γ	*SE*
Intercept	5.09***	0.18	5.07***	0.17	5.10***	0.16
Main effects						
Sex			0.10	0.25	0.14	0.25
Vitality			0.35**	0.12	0.38**	0.12
Personal fear of			–0.08	0.10	–0.07	0.10
invalidity						
Interaction effect						
Vitality × Personal					0.12	0.12
fear of invalidity						
Variance components						
Level 1 (σ^2^)	1.07		0.98		1.01	
Level 2 intercept (τ_00_)	0.19		0.13		0.08	
Model evaluation						
Model deviance	254.39		242.83		241.72	
*df*	3		6		7	
ΔDeviance			11.56**		1.11	

N = 85. Unstandardized coefficients (γ) and standard errors (SE) are shown. Sex was coded 1 = women (reference group), 2 = men. Deviance test is one-tailed. **p < 0.01. ***p < 0.001.

**TABLE 5:** Summary of the multilevel regression analysis for variables predicting manager-rated proactive behavior in Study 2.

	Null model		Main effect model		Model with interaction	
	γ	*SE*	γ	*SE*	γ	*SE*
Intercept	5.38***	0.17	5.49***	0.19	5.44***	0.16
Main effects						
Sex			–0.43	0.25	−0.52*	0.25
Vitality			0.18	0.12	0.11	0.12
Personal fear of			–0.02	0.10	–0.04	0.10
invalidity						
Interaction effect						
Vitality × Personal					−0.28*	0.12
fear of invalidity						
Variance components						
Level 1 (σ^2^)	1.01		0.97		0.98	
Level 2 intercept (τ_00_)	0.16		0.21		0.08	
Model evaluation						
Deviance	249.37		244.50		238.77	
*Df*	3		6		7	
ΔDeviance			4.87		5.73*	

11 department managers provided ratings for 85 subordinate employees’ proactive behavior. Unstandardized coefficients (γ) and standard errors (SE) are shown. Sex was coded 1 = women (reference group), 2 = men. Deviance test is one-tailed. *p < 0.05. ***p < 0.001.

**FIGURE 2:**
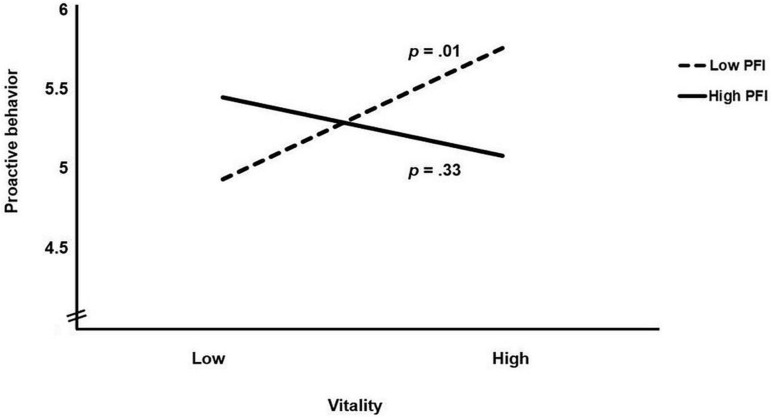
Moderating effect of personal fear of invalidity (PFI) on the relation between work-related vitality and manager ratings of employees’ proactive behavior in Study 2. “Low” and “high” represent values of 1 *SD* below and above the mean, respectively.

In the second hypothesis, we proposed that PFI moderated the positive relation between employees’ sense of vitality at work and their proactive behavior such that this positive relation was only present if PFI was low. The results did not support an interaction effect for self-rated proactive behavior, 95% CI [−0.12, 0.36]. However, the results supported an interactive effect between vitality and PFI for manager-rated proactive behavior, 95% CI [−0.51, −0.04]. The interaction effect is plotted in Figure 2, showing a crossover interaction. An analysis of simple slopes confirmed a positive relation between vitality and manager-rated proactive behavior for employees with a relatively low PFI (i.e., one *SD* below the mean), γ_*s*_ = 0.42, *SE* = 0.16, *p* = 0.01, but not for employees with a relatively high PFI (i.e., one *SD* above the mean), γ_*s*_ = −0.19, *SE* = 0.19, *p* = 0.33. Further, there was no evidence for a positive relation between vitality and proactive behavior for employees with an average PFI, γ_*s*_ = 0.11, *SE* = 0.12, *p* = 0.33. As such, our second hypothesis was only confirmed when proactive behavior was rated by the managers.

## Discussion

Proactive behavior is of increasing importance in today’s organizations and has emerged as a topic of great relevance for organizational research (Bindl and Parker, 2011; Parker and Bindl, 2016). As proactive behavior can increase the effectiveness of individual employees, teams, and organizations, a focal question is how to enhance proactivity in the workforce (Parker et al., 2006; Bindl and Parker, 2011; Strauss and Parker, 2018). Drawing on the model of proactive motivation (Parker et al., 2010) and Hobfoll’s (1989) Conservation of Resources Theory, we investigated whether employees’ sense of vitality at work is positively related to their proactive behavior, and whether this link is moderated by employees’ PFI (Thompson et al., 2001). In two studies, an experimental study (Study 1) and a cross-sectional field study (Study 2), we found empirical evidence for the predicted positive relation between employees’ sense of vitality and *self-rated* proactive behavior. As such, we complement earlier research on the link between positive affect and employees’ proactive behavior in three ways.

First, by using a randomized controlled design including manipulated work-related vitality (Study 1), we provide initial evidence that the link between vitality and proactivity is of a causal nature (e.g., Rubin, 1986). However, experimental follow-up studies using different manipulations of vitality to replicate the current effect are generally required to establish whether there is a causal link between vitality and proactivity (Spector, 2019). Moreover, on a note of caution, we examined situated behavioral preferences for proactivity rather than actual behavior in Study 1. This raises the concern whether the findings can be generalized to proactive behavior shown in the workplace. To resolve this uncertainty, field experiments conducted in organizations are required.

Second, our research supports the idea of a positive relation between on-the-job vitality and self-reported proactive *action*. This was demonstrated by relying on proactivity concepts that focus on the implementation of change/improvements in the work environment (e.g., Fuller et al., 2012). In contrast, earlier findings supporting a positive relation between vitality and proactivity addressed “employee voice” (Schmitt et al., 2017), which captures the “cooperative, *communication-based element* of organizational proactivity” (Thomas et al., 2010, p. 277, italics added). Moreover, the proactive behaviors we examined affect the “internal organizational environment” (Bledow and Frese, 2009; Parker and Collins, 2010, p. 636), whereas the positive link between vitality and proactivity found by Binyamin and Brender-Ilan (2018) is limited to an employee’s core tasks.

Third, by considering a moderating variable, our findings extend research on the link between positive affect and work-related proactivity (Fritz and Sonnentag, 2009; Bindl et al., 2012; Fay and Sonnentag, 2012; Sonnentag and Starzyk, 2015; Binyamin and Brender-Ilan, 2018). In the only previous study examining a moderator in the link between work-related vitality and proactivity, Schmitt et al. (2017) showed that the positive relation between vitality, measured in the morning, and end-of-day voice behavior was stronger among employees who reported being confident about succeeding in their job tasks. Our findings dovetail with those provided by Schmitt et al. (2017) in the sense that confidence (i.e., low PFI) seems to strengthen the link between vitality and proactivity, whereas feelings of anxiety or insecurity (i.e., high PFI) seem to reduce the likelihood of proactivity being shown by employees who experience a sense of vitality. The fact that the predicted positive relation between vitality and manager-rated proactivity in Study 2 was not supported is probably due to the moderation effect of PFI having a qualifying nature; namely, the relation was supported only for employees high in PFI.

Furthermore, the predicted moderation effect of PFI in the context of *self-rated* proactivity was not supported in either Study 1 or Study 2. The different patterns observed for self- and manager-rated proactivity can probably be linked to the fact that these ratings were essentially unrelated (*r* = −0.09, see Table 3). It would seem that self-ratings and manager ratings capture different aspects of employee proactivity. Indeed, Tornau and Frese (2013) note that unique perspectives and information can make employees and supervisors provide different – yet equally valid – answers to proactivity measures (see also Chan, 2009). In their meta-analytic review of proactivity concepts, including personal initiative and taking charge, Tornau and Frese (2013, p. 51) observed that proactivity concepts can be divided into two theoretical clusters; namely, a “personality cluster,” referring to proactivity as a trait, and a “behavior cluster,” referring to actual/observable behavioral manifestations of proactivity. Self-ratings of proactivity capture the extent to which employees regard themselves as being proactive individuals at work or the importance they attach to proactive behavior (i.e., they correspond to the personality cluster; Frese et al., 1997). In contrast, manager ratings capture observable proactive behavior in the workplace (i.e., part of the behavior cluster) that carries potentially negative social consequences and risks (e.g., Morrison and Phelps, 1999; Fay and Frese, 2001) and is, accordingly, a better proxy for actual proactive behavior (Tornau and Frese, 2013). This distinction could explain why the apprehension of the risks/negative consequences of making errors (PFI) only moderates the relation between vitality and proactive behavior when the latter is rated by managers. Overall, the results only support a positive relation between vitality and observable manager-rated proactive behavior when employees’ fear of invalidity is low. Our findings indicate that, when mentally imagining how one *would* behave (Study 1) or when self-reporting proactive behavior (Study 2), employees both low and high in PFI are likely to evaluate themselves as proactive, provided their sense of vitality at work is high.

In this context, it is important to note that the observed main effect of work-related vitality on proactivity in our experimental study (Study 1) can primarily be linked to the low-vitality condition. As shown by the manipulation check, in terms of recalled experienced vitality, employees in the control condition differed more substantially from those in the low-vitality condition than from those in the high-vitality condition. This finding could explain the lack of a significant difference in proactivity between the high-vitality condition and the control condition. The relatively high mean in the control condition could indicate, given that the participants in the control condition were asked to recall a typical workday, that experiencing a sense of vitality is the default state in the population of employees. This assumption is supported by the high mean for vitality observed in Study 2 (see also Binyamin and Brender-Ilan, 2018).

### Implications

As proactive behavior enhances all aspects of employees’ job performance (Bindl and Parker, 2011), our findings support the view that employees’ sense of vitality is important to organizations because it facilitates “maximizing work performance as well as worker health and well-being” (i.e., sustainable performance; De Jonge and Peeters, 2019, p. 1). Our findings confirm a central assertion of the model of proactive motivation – that activated positive affect is an antecedent of employees’ proactivity (Parker et al., 2010). In terms of this tenet, the initial evidence presented for a causal positive effect of employees’ sense of vitality on behavioral preferences for proactivity (see Study 1) is of substantial value (Bindl et al., 2012).

Our findings indicate that employees who experience a sense of vitality at work are unlikely to demonstrate additional observable proactive behavior if they have a strong inclination toward fearing making mistakes. According to the model of proactive motivation, personality and individual-difference variables are important to understand the occurrence of proactivity in the workplace (Parker et al., 2010; Wu et al., 2013). Our findings provide initial evidence for PFI to be added to the list of previously identified affective traits (trait affectivity and neuroticism; see Wu et al., 2013). As shown in Study 2, this individual-difference variable may counteract the effect of vitality on proactive behavior. As such, showing the moderating role of PFI confirms the important role of negative affect in understanding the process of proactive goal pursuit (Bindl, 2019). Specifically, our finding dovetails with the empirically supported view that while positive affect motivates the proactive implementation of change (Parker et al., 2010; Sonnentag and Starzyk, 2015), the discrete emotion of fear may eventually prevent this from happening (Bindl, 2019).

Based on the observed moderation effect of PFI, we would recommend that organizations aiming to foster their employees’ proactivity use two strategies: creating a work environment that conduces to employees’ experience of vitality and recruiting individuals with a low PFI. Our findings suggest combining these strategies rather than using either of them (Parker et al., 2006). An alternative to recruiting may be creating a work climate that conveys the message that socially risky behaviors will not lead to adverse reactions (Edmondson and Lei, 2014). In further support of this recommendation, a field study conducted by Deng et al. (2019) has revealed that, on the group level, perceptions of psychological safety were positively associated with proactive voice behavior through reduced levels of fear of failure.

### Strengths and Limitations

A strength of our research is using two different samples, methods/designs, and concepts and measures of proactive behavior to investigate the link between vitality and proactivity at work. The consistent finding of a positive relation between work-related vitality and self-reported proactive behavior found through the two different methods used to test our hypotheses allows us to claim high validity – both internally (Study 1) and externally (Study 2). Furthermore, we found consistent evidence for a positive relation between work-related vitality and self-reported proactivity across two proactivity concepts (i.e., personal initiative and taking charge) and two measures (i.e., a situational judgment test and a rating scale), which strengthens the robustness of this finding.

Our research also has limitations, including the sample size in Study 2. Given that it is difficult to detect moderation effects in field studies (McClelland and Judd, 1993), it is possible that the statistical power was insufficient to detect the predicted moderation effect for *self-rated proactivity*. Another limitation is that our studies did not allow testing of reversed causation. It is likely that proactive behavior will also increase the sense of vitality because such behavior is conducive to fulfilling employees’ basic psychological needs (i.e., the needs for autonomy, competence, and relatedness; Strauss and Parker, 2014). The fulfillment of these needs, in turn, amounts to individuals’ energizing nutriments that enhance vitality (Ryan and Deci, 2008; Wörtler et al., 2020). Indeed, Cangiano et al. (2019) have shown that employees’ daily proactive behavior is positively related to end-of-day vitality through enhanced levels of self-perceived competence.

### Future Research and Conclusions

As proactivity can have undesirable individual-level consequences (Parker et al., 2019), proactive behaviors that might carry even greater social risk than the ones we considered, such as voicing and advocating radical innovative ideas or pointing out critical issues to superiors in order to affect the strategy of the organization (see Janssen et al., 2004; Parker and Collins, 2010; Sijbom et al., 2015), could well vary as a function of employees’ PFI. We would therefore encourage PFI to be included in future studies on proactive behavior. Future studies could also replicate the investigated moderation effect of PFI on the relation between vitality and proactivity, specifically focusing on exploring the roles of self-rated and manager-rated proactive behavior. For example, participants could be instructed to take the manager’s perspective when rating their own proactivity. Schoorman and Mayer (2008) found a much higher correlation between self-ratings and supervisor ratings of job performance when employees were asked to adopt their supervisor’s perspective compared with when this instruction was not given. A similar pattern may be found for proactive behavior. In addition, the role of managers’ behavior could be examined. For example, the interaction between vitality and PFI with regard to manager-rated proactivity may be particularly noticeable when managers blame employees for their errors (Cangiano et al., 2019), whereas employees’ perceptions of managers’ openness to suggestions and ideas may attenuate or eliminate that interaction (Lebel, 2016).

## Conclusion

In conclusion, the current research has shown that employees tend to report an enhanced proclivity for proactive behavior when they experience a strong sense of vitality at work. However, employees’ sense of vitality is not necessarily associated with observable proactive behavior. It is only when employees experiencing a sense of vitality at work are not prone to fearing the risks/negative consequences of making errors that they are more likely to show observable proactive behavior in an organization.

## Data Availability Statement

The raw data supporting the conclusions of this article will be made available by the authors, without undue reservation, to any qualified researcher.

## Ethics Statement

The studies involving human participants were reviewed and approved by the Ethics Committee of Psychology of the University of Groningen. All participants provided active informed consent.

## Author Contributions

BW, NV, and DB contributed to the conception and to the design of the studies. JM and BW collected the data. BW performed the statistical analyses and wrote the first draft of the manuscript. BW and NV developed the manuscript further. All authors contributed to the manuscript revision, read and approved the submitted version.

## Conflict of Interest

The authors declare that the research was conducted in the absence of any commercial or financial relationships that could be construed as a potential conflict of interest.

## Appendix

### Instructions for the Participants to Manipulate Work-Related Vitality in Study 1

#### High-Vitality Condition

Please recall a workday on which you experienced a sense of vitality at work. By that, we mean that you had a lot of energy and enthusiasm at work. After that, please relive that workday on which you experienced a sense of vitality at work, and describe as vividly and in as much detail as possible what happened on that day at work, what you thought, what you did, etc.

#### Low-Vitality Condition

Please recall a workday on which you did not experience a sense of vitality at work. By that, we mean that you lacked energy and enthusiasm at work. After that, please relive that workday on which you did not experience a sense of vitality at work, and describe as vividly and in as much detail as possible what happened on that day at work, what you thought, what you did, etc.

#### Control Condition

Please recall a typical workday. After that, please relive that typical workday, and describe as vividly and in as much detail as possible what happened on that day at work, what you thought, what you did, etc.

## References

[B1] Bateman T. S. Crant J. M. (1993). The proactive component of organizational behavior: a measure and correlates. *J. Organ. Behav.* 14 103–118. 10.4324/978131579711310.1002/job.4030140202

[B2] Belschak F. D. Den Hartog D. N. (2010). Pro-self, prosocial, and pro-organizational foci of proactive behaviour: differential antecedents and consequences. *J. Occupat. Organ. Psychol.* 83 475–498. 10.1348/096317909X439208 30467716

[B3] Bergeron D. M. (2007). The potential paradox of organizational citizenship behavior: good citizens at what cost? *Acad. Manag. Rev.* 32 1078–1095. 10.5465/amr.2007.26585791

[B4] Bindl U. K. (2019). Work-related proactivity through the lens of narrative: investigating emotional journeys in the process of making things happen. *Hum. Relat.* 72 615–645. 10.1177/0018726718778086

[B5] Bindl U. K. Parker S. K. (2011). “Proactive work behavior: forward-thinking and change-oriented action in organizations,” in *APA Handbook of Industrial and Organizational Psychology*, Vol. 2 ed. Zedeck S. (Washington, DC: American Psychological Association), 567–598. 10.1037/12170-019

[B6] Bindl U. K. Parker S. K. Totterdell P. Hagger-Johnson G. (2012). Fuel of the self-starter: how mood relates to proactive goal regulation. *J. Appl. Psychol.* 97 134–150. 10.1037/a0024368 21744938

[B7] Binyamin G. Brender-Ilan Y. (2018). Leaders’ language and employee proactivity: enhancing psychological meaningfulness and vitality. *Eur. Manag. J.* 36 463–473. 10.1016/j.emj.2017.09.004

[B8] Bledow R. Frese M. (2009). A situational judgment test of personal initiative and its relationship to performance. *Person. Psychol.* 62 229–258. 10.1111/j.1744-6570.2009.01137.x

[B9] Bolino M. C. Valcea S. Harvey J. (2010). Employee, manage thyself: the potentially negative implications of expecting employees to behave proactively. *J. Occup. Organ. Psychol.* 83 325–346. 10.1348/096317910X493134 30467716

[B10] Bostic T. J. McGartland Rubio D. Hood M. (2000). A validation of the subjective vitality scale using structural equation modeling. *Soc. Indicat. Res.* 52 313–324. 10.1023/A:1007136110218

[B11] Buhrmester M. Kwang T. Gosling S. D. (2011). Amazon’s Mechanical Turk: a new source of inexpensive, yet high-quality, data? *Perspect. Psychol. Sci.* 6 3–5. 10.1177/1745691610393980 26162106

[B12] Calderwood C. Bennett A. A. Gabriel A. S. Trougakos J. P. Dahling J. J. (2018). Too anxious to help? Off-job affective rumination as a linking mechanism between work anxiety and helping. *J. Occup. Organ. Psychol.* 91 681–687. 10.1111/joop.12220

[B13] Cangiano F. Parker S. K. Yeo G. B. (2019). Does daily proactivity affect well-being? The moderating role of punitive supervision. *J. Organ. Behav.* 40 59–72. 10.1002/job.2321

[B14] Chan D. (2009). “So why ask me? are self-report data really that bad?,” in *Statistical and Methodological Myths and Urban Legends: Doctrine Verity and Fable in the Organizational and Social Sciences*, eds Lance C. E. Vandenberg R. J. (New York, NY: Routledge), 309–336.

[B15] Christian M. S. Garza A. S. Slaughter J. E. (2011). Work engagement: a quantitative review and test of its relations with task and contextual performance. *Pers. Psychol.* 64 89–136. 10.1111/j.1744-6570.2010.01203.x

[B16] Cohen J. (1992). A power primer. *Psychol. Bull.* 112 155–159. 10.1037/0033-2909.112.1.155 19565683

[B17] Cohen J. Cohen P. West S. G. Aiken L. S. (2003). *Applied Multiple Regression/Correlation Analysis for the Behavioral Sciences*, 3rd Edn, New Jersey: Lawrence Erlbaum Associates Publishers.

[B18] Crandall C. S. Sherman J. W. (2016). On the scientific superiority of conceptual replications for scientific progress. *J. Exper. Soc. Psychol.* 66 93–99. 10.1016/j.jesp.2015.10.002

[B19] Crant J. M. (2000). Proactive behavior in organizations. *J. Manag.* 26 435–462. 10.1177/014920630002600304

[B20] De Jonge J. Peeters M. C. W. (2019). The vital worker: towards sustainable performance at work. *Intern. J. Environ. Res. Public Health* 16:910. 10.3390/ijerph16060910 30871247 PMC6466057

[B21] Deng H. Leung K. Lam C. K. Huang X. (2019). Slacking off in comfort: a dual-pathway model for psychological safety climate. *J. Manag.* 45 1114–1144. 10.1177/0149206317693083

[B22] Edmondson A. C. Lei Z. (2014). Psychological safety: the history, renaissance, and future of an interpersonal construct. *Annu. Rev. Organ. Psychol. Organ. Behav.* 1 23–43. 10.1146/annurev-orgpsych-031413-091305

[B23] Fay D. Frese M. (2001). The concept of personal initiative: an overview of validity studies. *Hum. Perform.* 14 97–124. 10.1207/S15327043HUP1401_06

[B24] Fay D. Sonnentag S. (2012). Within-person fluctuations of proactive behavior: how affect and experienced competence regulate work behavior. *Hum. Perform.* 25 72–93. 10.1080/08959285.2011.631647

[B25] Fennis B. M. Stroebe W. (2010). *The Psychology of Advertising.* London: Psychology Press.

[B26] Finch W. H. Bolin J. E. Kelley K. (2014). *Multilevel Modeling Using R.* Boca Raton, FL: CRC Press.

[B27] Frese M. Fay D. Hilburger T. Leng K. Tag A. (1997). The concept of personal initiative: operationalization, reliability and validity in two German samples. *J. Occup. Organ. Psychol.* 70 139–161. 10.1111/j.2044-8325.1997.tb00639.x

[B28] Fritz C. Sonnentag S. (2009). Antecedents of day-level proactive behavior: a look at job stressors and positive affect during the workday. *J. Manag.* 35 94–111. 10.1177/0149206307308911

[B29] Fuller B. Jr. Marler L. E. (2009). Change driven by nature: a meta-analytic review of the proactive personality literature. *J. Vocat. Behav.* 75 329–345. 10.1016/j.jvb.2009.05.008

[B30] Fuller B. Jr. Marler L. E. Hester K. (2012). Bridge building within the province of proactivity. *J. Organ. Behav.* 33 1053–1070. 10.1002/job.1780

[B31] Galinsky A. Gruenfeld D. Magee J. (2003). From power to action. *J. Pers. Soc. Psychol.* 85 453–466. 10.1037/0022-3514.85.3.453 14498782

[B32] Grant A. M. Ashford S. J. (2008). The dynamics of proactivity at work. *Res. Organ. Behav.* 28 3–34. 10.1016/j.riob.2008.04.002

[B33] Griffin M. A. Neal A. Parker S. K. (2007). A new model of work role performance: positive behavior in uncertain and interdependent contexts. *Acad. Manag. J.* 50 327–347. 10.5465/AMJ.2007.24634438

[B34] Hobfoll S. E. (1989). Conservation of resources: a new attempt at conceptualizing stress. *Am. Psychol.* 44 513–524. 10.1037/0003-066X.44.3.513 2648906

[B35] Ilies R. Scott B. A. Judge T. A. (2006). The interactive effects of personal traits and experienced states on intraindividual patterns of citizenship behavior. *Acad. Manag. J.* 49 561–575. 10.5465/amj.2006.21794672

[B36] Janssen O. Van de Vliert E. West M. (2004). The bright and dark sides of individual and group innovation: a special issue introduction. *J. Organ. Behav.* 25 129–145. 10.1002/job.242

[B37] Keith M. G. Tay L. Harms P. D. (2017). Systems perspective of Amazon Mechanical Turk for organizational research: review and recommendations. *Front. Psychol.* 8:1359. 10.3389/fpsyg.2017.01359 28848474 PMC5550837

[B38] Kish-Gephart J. J. Detert J. R. Treviño L. K. Edmondson A. C. (2009). Silenced by fear: the nature, sources, and consequences of fear at work. *Res. Organ. Behav.* 29 163–193. 10.1016/j.riob.2009.07.002

[B39] Landers R. N. Behrend T. S. (2015). An inconvenient truth: arbitrary distinctions between organizational, Mechanical Turk, and other convenience samples. *Industr. Organ. Psychol.* 8 142–164. 10.1017/iop.2015.13

[B40] Lebel R. D. (2016). Overcoming the fear factor: how perceptions of supervisor openness lead employees to speak up when fearing external threat. *Organ. Behav. Hum. Decis. Process.* 135 10–21. 10.1016/j.obhdp.2016.05.001

[B41] Li R. Zhang Z.-Y. Tian X.-M. (2016). Can self-sacrificial leadership promote subordinate taking charge? The mediating role of organizational identification and the moderating role of risk aversion. *J. Organ. Behav.* 37 758–781. 10.1002/job.2068

[B42] MacKinnon D. P. Coxe S. Baraldi A. N. (2012). Guidelines for the investigation of mediating variables in business research. *J. Bus. Psychol.* 27 1–14. 10.1007/s10869-011-9248-z 25237213 PMC4165346

[B43] McAllister D. J. Kamdar D. Morrison E. W. Turban D. B. (2007). Disentangling role perceptions: how perceived role breadth, discretion, instrumentality, and efficacy relate to helping and taking charge. *J. Appl. Psychol.* 92 1200–1211. 10.1037/0021-9010.92.5.1200 17845080

[B44] McCarthy J. M. Trougakos J. P. Cheng B. H. (2016). Are anxious workers less productive workers? It depends on the quality of social exchange. *J. Appl. Psychol.* 101 279–291. 10.1037/apl0000044 26375962

[B45] McClelland G. H. Judd C. M. (1993). Statistical difficulties of detecting interactions and moderator effects. *Psychol. Bull.* 114 376–390. 10.1037/0033-2909.114.2.376 8416037

[B46] Meade A. W. Craig S. B. (2012). Identifying careless responses in survey data. *Psychol. Methods* 17 437–455. 10.1037/a0028085 22506584

[B47] Morrison E. W. Phelps C. C. (1999). Taking charge at work: extrarole efforts to initiate workplace change. *Acad. Manag. J.* 42 403–419. 10.5465/257011

[B48] Nix G. A. Ryan R. M. Manly J. B. Deci E. L. (1999). Revitalization through self-regulation: the effects of autonomous and controlled motivation on happiness and vitality. *J. Exper. Soc. Psychol.* 35 266–284. 10.1006/jesp.1999.1382

[B49] Parker S. K. Bindl U. K. (2016). “Proactivity at work: a big picture perspective on a construct that matters,” in *Proactivity at Work: Making Things Happen in Organizations*, eds Parker S. K. Bindl U. K. (Abingdon: Routledge), 1–20.

[B50] Parker S. K. Bindl U. K. Strauss K. (2010). Making things happen: a model of proactive motivation. *J. Manag.* 36 827–856. 10.1177/0149206310363732

[B51] Parker S. K. Collins C. G. (2010). Taking stock: integrating and differentiating multiple proactive behaviors. *J. Manag.* 36 633–662. 10.1177/0149206308321554

[B52] Parker S. K. Wang Y. Liao J. (2019). When is proactivity wise? A review of factors that influence the individual outcomes of proactive behavior. *Annu. Rev. Organ. Psychol. Organ. Behav.* 6 221–248. 10.1146/annurev-orgpsych-012218-015302

[B53] Parker S. K. Williams H. M. Turner N. (2006). Modeling the antecedents of proactive behavior at work. *J. Appl. Psychol.* 91 636–652. 10.1037/0021-9010.91.3.636 16737360

[B54] Paulhus D. L. (2002). “Socially desirable responding: the evolution of a construct,” in *The Role of Constructs in Psychological and Educational Measurement*, eds Braun H. I. Jackson D. N. Wiley D. E. (New Jersey: Lawrence Erlbaum Associates Publishers), 49–69.

[B55] Peer E. Vosgerau J. Acquisti A. (2014). Reputation as a sufficient condition for data quality on Amazon Mechanical Turk. *Behav. Res. Methods* 46 1023–1031. 10.3758/s13428-013-0434-y 24356996

[B56] Pinheiro J. Bates D. DebRoy S. Sarkar D., and R Core Team (2020). *nlme: Linear and Nonlinear Mixed Effects Models. R Package Version 3.1-147.* Available online at: https://CRAN.R-project.org/package=nlme 10.3758/s13428-013-0434-y 24356996

[B57] Porath C. Spreitzer G. Gibson C. Garnett F. G. (2012). Thriving a work: toward its measurement, construct validation, and theoretical refinement. *J. Organ. Behav.* 33 250–275. 10.1002/job.756

[B58] R Core Team (2019). *R: A Language and Environment for Statistical Computing.* Vienna: R Foundation for Statistical Computing.

[B59] Rubin D. B. (1986). Comment: which ifs have causal answers. *J. Am. Statist. Assoc.* 81 961–962. 10.1080/01621459.1986.10478355

[B60] Ryan R. M. Bernstein J. (2004). “Vitality/Zest/enthusiasm/vigor/energy,” in *Character Strengths and Virtues: A Handbook and Classification*, eds Petersen C. Seligman M. E. P. (Oxford: Oxford University Press), 273–289.

[B61] Ryan R. M. Deci E. L. (2008). From ego depletion to vitality: theory and findings concerning the facilitation of energy available to the self. *Soc. Pers. Psychol. Compass* 2 702–717. 10.1111/j.1751-9004.2008.00098.x

[B62] Ryan R. M. Frederick C. (1997). On energy, personality, and health: subjective vitality as a dynamic reflection of well-being. *J. Pers.* 65 529–565. 10.1111/j.1467-6494.1997.tb00326.x 9327588

[B63] Ryan R. M. Weinstein N. Bernstein J. Brown K. W. Mistretta L. Gagné M. (2010). Vitalizing effects of being outdoors and in nature. *J. Environ. Psychol.* 30 159–168. 10.1016/j.jenvp.2009.10.009

[B64] Salanova M. Schaufeli W. B. (2008). A cross-national study of work engagement as a mediator between job resources and proactive behaviour. *Intern. J. Hum. Resour. Manag.* 19 116–131. 10.1080/09585190701763982

[B65] Schaufeli W. B. Salanova M. González-Romá V. Bakker A. B. (2002). The measurement of engagement and burnout: a two sample confirmatory factor analytic approach. *J. Happ. Stud.* 3 71–92. 10.1023/A:1015630930326

[B66] Schmitt A. Belschak F. D. Den Hartog D. N. (2017). Feeling vital after a good night’s sleep: the interplay of energetic resources and self-efficacy for daily proactivity. *J. Occup. Health Psychol.* 22 443–454. 10.1037/ocp0000041 27123889

[B67] Schmitt A. Den Hartog D. N. Belschak F. D. (2016). Transformational leadership and proactive work behaviour: a moderated mediation model including work engagement and job strain. *J. Occup. Organ. Psychol.* 89 588–610. 10.1111/joop.12143

[B68] Schoorman F. D. Mayer R. C. (2008). The value of common perspectives in self-reported appraisals: you get what you ask for. *Organ. Res. Methods* 11 148–159. 10.1177/1094428107307168

[B69] Sijbom R. B. L. Janssen O. Van Yperen N. W. (2015). How to get radical creative ideas into a leader’s mind? Leader’s achievement goals and subordinates’ voice of creative ideas. *Eur. J. Work Organ. Psychol.* 24 279–296. 10.1080/1359432X.2014.892480

[B70] Snijders T. A. B. Bosker R. J. (2012). *Multilevel Analysis: An Introduction to Basic and Advanced Multilevel Modeling*, 2nd Edn, New York, NY: SAGE Publications Ltd.

[B71] Sonnentag S. Starzyk A. (2015). Perceived prosocial impact, perceived situational constraints, and proactive work behavior: looking at two distinct affective pathways. *J. Organ. Behav.* 36 806–824. 10.1002/job.2005

[B72] Spector P. E. (2019). Do not cross me: optimizing the use of cross-sectional designs. *J. Bus. Psychol.* 34 125–137. 10.1007/s10869-018-09613-8

[B73] Strauss K. Parker S. K. (2014). “Effective and sustained proactivity in the workplace: a self-determination theory perspective,” in *Oxford Handbook Of Work Engagement Motivation and Self-Determination Theory*, ed. Gagne M. (Oxford: Oxford University Press), 50–71. 10.1093/oxfordhb/9780199794911.013.007

[B74] Strauss K. Parker S. K. (2018). Intervening to enhance proactivity in organizations: improving the present or changing the future. *J. Manag.* 44 1250–1278. 10.1177/0149206315602531

[B75] Thomas J. P. Whitman D. S. Viswesvaran C. (2010). Employee proactivity in organizations: a comparative meta-analysis of emergent proactive constructs. *J. Occup. Organ. Psychol.* 83 275–300. 10.1348/096317910X502359 30467716

[B76] Thompson M. M. Naccarato M. E. Parker K. C. H. Moskowitz G. B. (2001). “The personal need for structure and personal fear of invalidity measures: historical perspectives, current applications, and future directions,” in *Cognitive Social Psychology: The Princeton Symposium on the Legacy and Future of Social Cognition*, ed. Moskowitz G. B. (New Jersey: Lawrence Erlbaum Associates), 19–39.

[B77] Thompson M. M. Zanna M. P. (1995). The conflicted individual: personality-based and domain-specific antecedents of ambivalent social attitudes. *J. Pers.* 63 259–288. 10.1111/j.1467-6494.1995.tb00810.x 7782994

[B78] Tornau K. Frese M. (2013). Construct clean-up in proactivity research: a meta-analysis on the nomological net of work-related proactivity concepts and their incremental validities. *Appl. Psychol.* 62 44–96. 10.1111/j.1464-0597.2012.00514.x

[B79] Wörtler B. Van Yperen N. W. Barelds D. P. H. (2020). Do individual differences in need strength moderate the relations between basic psychological need satisfaction and organizational citizenship behavior? *Motiv. Emot.* 44 315–328. 10.1007/s11031-019-09775-9

[B80] Wu C.-H. Parker S. K. (2017). The role of leader support in facilitating proactive work behavior. *J. Manag.* 43 1025–1049. 10.1177/0149206314544745

[B81] Wu C.-H. Parker S. K. Bindl U. K. (2013). “Who is proactive and why? Unpacking individual differences in employee proactivity,” in *Advances in Positive Organizational Psychology*, Vol. 1 ed. Bakker A. B. (Bingley: Emerald Group Publishing), 261–280. 10.1108/s2046-410x(2013)0000001014

[B82] Wu C.-H. Parker S. K. Wu L.-Z. Lee C. (2018). When and why people engage in different forms of proactive behavior: interactive effects of self-construals and work characteristics. *Acad. Manag. J.* 61 293–323. 10.5465/amj.2013.1064

